# TIE2 Induces Breast Cancer Cell Dormancy and Inhibits the Development of Osteolytic Bone Metastases

**DOI:** 10.3390/cancers12040868

**Published:** 2020-04-03

**Authors:** Florian Drescher, Patricia Juárez, Danna L. Arellano, Nicolás Serafín-Higuera, Felipe Olvera-Rodriguez, Samanta Jiménez, Alexei F. Licea-Navarro, Pierrick G.J. Fournier

**Affiliations:** 1 Biomedical Innovation Department, Centro de Investigación Científica y de Educación Superior de Ensenada (CICESE), Ensenada, Baja California 22860, Mexico; drescher@cicese.edu.mx (F.D.); pjuarez@cicese.mx (P.J.); darellan@cicese.edu.mx (D.L.A.); mjimenez@cicese.edu.mx (S.J.); alicea@cicese.mx (A.F.L.-N.); 2 Posgrado en Ciencias de la Vida, Centro de Investigación Científica y de Educación Superior de Ensenada (CICESE), Ensenada, Baja California 22860, Mexico; 3 Unidad de Ciencias de la Salud, Facultad de Odontología, Universidad Autónoma de Baja California, Mexicali, Baja California 21040, Mexico; nserafin@uabc.edu.mx; 4 Departamento de Biología Molecular y Bioprocesos, Instituto de Biotecnología Universidad Nacional Autónoma de Mexico, Cuernavaca, Morelos 62210, Mexico; folvera@ibt.unma.mx

**Keywords:** breast cancer, bone metastasis, dormancy, TIE2, chemotherapy resistance, cancer relapse

## Abstract

Breast cancer (BCa) cells disseminating to the bone can remain dormant and resistant to treatments for many years until relapsing as bone metastases. The tyrosine kinase receptor TIE2 induces the dormancy of hematopoietic stem cells, and could also induce the dormancy of BCa cells. However, TIE2 is also a target for anti-angiogenic treatments in ongoing clinical trials, and its inhibition could then restart the proliferation of dormant BCa cells in bone. In this study, we used a combination of patient data, in vitro, and in vivo models to investigate the effect of TIE2 in the dormancy of bone metastases. In BCa patients, we found that a higher *TIE2* expression is associated with an increased time to metastases and survival. In vitro, TIE2 decreased cell proliferation as it increased the expression of cyclin-dependent kinase inhibitors *CDKN1A* and *CDKN1B* and arrested cells in the G_0_/G_1_ phase. Expression of *TIE2* also increased the resistance to the chemotherapeutic 5-Fluorouracil. In mice, *TIE2* expression reduced tumor growth and the formation of osteolytic bone metastasis. Together, these results show that TIE2 is sufficient to induce dormancy in vitro and in vivo, and could be a useful prognostic marker for patients. Our data also suggest being cautious when using TIE2 inhibitors in the clinic, as they could awaken dormant disseminated tumor cells.

## 1. Introduction

Breast cancer (BCa) is the most common cancer in women worldwide [1]. BCa cells have a propensity to home to the bone, causing bone metastases in at least 75% of patients with advanced-stage BCa [2]. The development of these bone metastases can occur years or decades after the treatment of the primary tumor [3]. During this time, although patients appear to be cancer-free, disseminated tumor cells (DTCs) are lodged in their bone marrow, where they remain in a stage of dormancy [4]. Cellular dormancy is characterized by reduced metabolism and cell growth arrest that render them resistant to chemo- and radiotherapy [5]. A similar phenomenon occurs in patients with prostate cancer (PCa) that also favors bone as a site of metastasis [2,6]. When dormant DTCs awaken, bone metastases are formed that will eventually lead to a fatal outcome, since the available treatments do not cure bone metastases and only slow down their progression [7]. Thus, it is essential to identify the mechanisms that regulate dormancy and can serve as targets for future treatments.

In the last two decades, research showed that the bone microenvironment plays a crucial role in the development of bone metastases. As cancer cells enter the bone marrow, they are attracted to the osteoblastic hematopoietic stem cell (HSC) niche by local factors, such as the chemokine CXCL12 and the attachment factor annexin II that normally retain HSCs in this niche [8,9,10]. Subsequently, factors from the microenvironment such as osteoblast-derived Bone Morphogenetic Protein 7 (BMP7) and Growth Arrest Specific 6 (GAS6) will induce the dormancy of PCa cells, while osteoblast-derived Jagged 1 (JAG1) and hypoxia induce the dormancy of BCa cells [11,12,13,14]. Like with the homing, the same mechanisms that induce the dormancy of DTCs induce the dormancy of HSCs in the bone marrow [15,16,17,18].

Another factor that regulates HSC behavior and dormancy is TIE2 (also named TEK or CD202b), a tyrosine kinase receptor for angiopoietin-1 (ANGPT1) and -2 (ANGPT2). The ANGPT1/TIE2 axis is critical for HSCs maintenance as it promotes their self-renewal activity and long-term reconstitution in lethally irradiated mice [19]. This effect is due to the induction of quiescence or dormancy in HSCs both in vitro and in vivo [19]. Similar to other factors that induce HSC dormancy, TIE2 activation also induces dormancy in PC-3 PCa cells and increases their resistance to the chemotherapeutic drug Cabazitaxel [20]. This suggests that TIE2-induced dormancy could protect DTCs in the bone during chemotherapy, and these cells may re-awaken at a later time point to form bone metastasis.

In addition, TIE2 is a cornerstone of vessel remodeling and angiogenesis during normal physiological conditions and tumor growth [21]. In recent years, anti-angiogenic therapies that target TIE2 or its ligands (ANGPT1 and ANGPT2) have been developed and are being tested in clinical trials in combination with chemotherapy [22,23]. Therefore, although anti-ANGPT/TIE2 therapies could be efficient at inhibiting tumor angiogenesis, they may reverse the dormancy of DTCs induced by TIE2 in the bone marrow and support the development of bone metastases. For these reasons, our goal was to understand further the role of TIE2 in the dormancy of BCa cells and bone metastases, combining patient data, as well as in vitro and in vivo models.

In this study, we found that *TIE2* expression in the primary tumor of breast cancer patients is associated with a longer time until metastases or relapse, and prolonged overall survival. At the same time, *TIE2* expression appeared to confer a growth disadvantage to both tumors in patients, and BCa and PCa cells in culture, due to the induction of dormancy. In vitro, we demonstrated that *TIE2* expression alone was sufficient to induce dormancy, reducing cell proliferation and increasing chemotherapeutic resistance of MCF-7 cells. Consequently, in vivo, the induction of *TIE2* expression reduced the growth of the primary tumor and the development of osteolytic bone metastases.

## 2. Results

### 2.1. High TIE2 Expression Correlates with Increased Time to the Development of Metastases and Survival of BCa Patients

**Figure 1:**
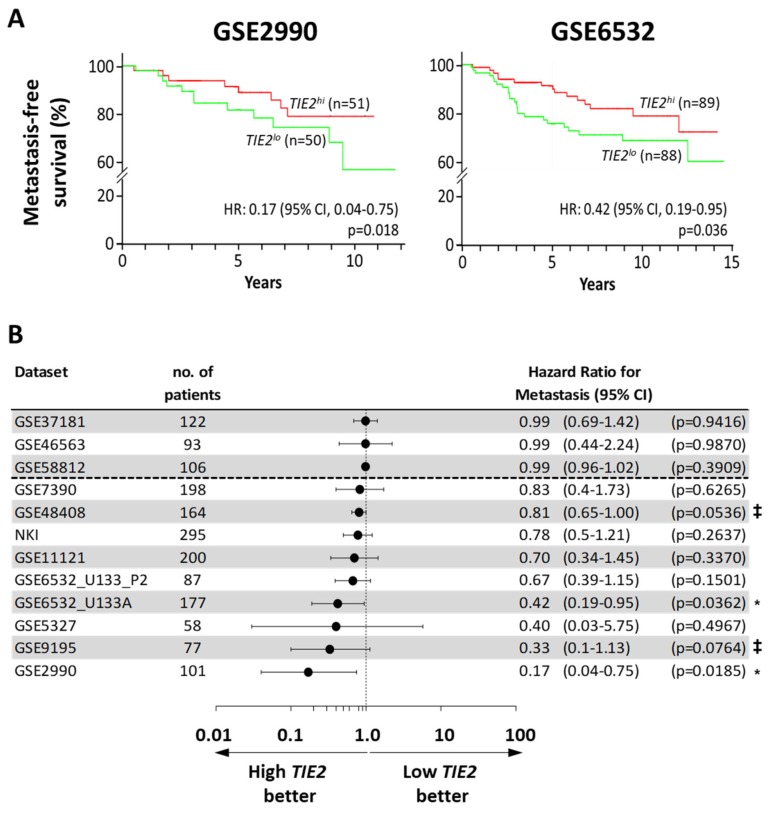
High *TIE2* expression in the primary tumor is associated with an increased time to the detection of metastases in breast cancer patients. Analysis of metastasis-free survival using the PROGgene database. The median *TIE2* mRNA level in the primary tumor was taken as a bifurcation point. Results are presented as (**A**) Kaplan–Meier plots for the Sotiriou (GSE2990) and Loi datasets (GSE6532), or as (**B**) a forest plot, indicating the overall hazard ratio (HR) for metastasis-occurrence and 95% confidence interval (CI). Survival analysis was performed using a log-rank test, ^‡^ *p* < 0.078, and * *p* < 0.05.

Since previous research has shown that TIE2 could directly induce the dormancy of hematopoietic stem cells and prostate cancer cells in vitro, we wanted to investigate first the clinical relevance of *TIE2* expression in cancer progression [19,20]. We compared the clinical outcome between BCa patients with a higher and lower expression of *TIE2* in their primary tumor, using the PROGgene database [24]. We found 12 datasets or cohorts of patients with information on the time to development of metastases. A higher expression of *TIE2* was significantly associated with an increased metastasis-free survival time in the datasets GSE2990 (HR = 0.17, 95% CI = 0.04 to 0.75; *p* = 0.018) and GSE5237 (HR = 0.42, 95% CI = 0.19 to 0.95; *p* = 0.036) (Figure 1A) [25,26]. Additionally, among the other 10 datasets analyzed, higher *TIE2* expression was nearly-significantly associated to an increased time before the development of metastases (*p* < 0.078) in the datasets GSE9195 (HR = 0.33, 95% CI = 0.10 to 1.13; *p* = 0.076) and GSE48408 (HR = 0.81, 95% CI = 0.65 to 1.00; *p* = 0.054) (Figure 1B) [27,28]. Overall, in 9 out of 12 (75%) of the datasets, comparing *TIE2^hi^* against *TIE2^lo^* patients, the hazard ratio was inferior to 0.85, indicating that a higher expression of *TIE2* in the primary tumor of BCa patients is associated with a longer time until the development of metastases.

**Figure 2:**
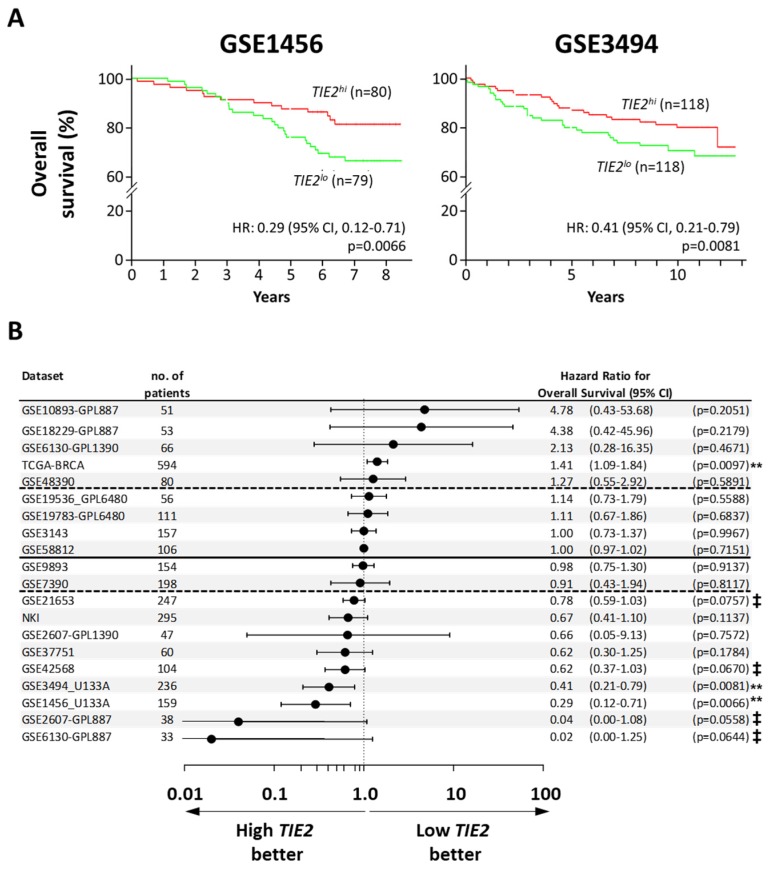
High expression of *TIE2* in the primary tumor of breast cancer patients is associated with increased overall survival. Analysis of overall survival using the PROGgene database. The median *TIE2* mRNA level in the primary tumor was taken as a bifurcation point. Results are presented as (**A**) Kaplan–Meyer plots for the Pawitan (GSE1456) and Miller datasets (GSE3494), or as (**B**) a forest plot, indicating the overall hazard ratio (HR) for metastasis-occurrence and 95% confidence interval (CI). The horizontal line divides datasets with a HR higher or lower than 1, the dotted lines separate the datasets with a HR lower than 0.85 and higher than 1.15. Survival analysis was performed using a log-rank test, ^‡^ *p* < 0.078, and ** *p* < 0.01.

When assessing relapse-free survival, we found 26 datasets reporting this outcome. Among them, the hazard ratio was inferior to 0.85 in 17 datasets (65%), and a higher *TIE2* expression was significantly associated to a longer time to relapse in 3 datasets: GSE1456 (HR = 0.21, 95% CI = 0.08 to 0.53, *p* = 0.001), GSE17705 (HR = 0.3, 95% CI = 0.12 to 0.76; *p* = 0.011), and GSE4922 (HR = 0.53, 95% CI = 0.32 to 0.88; *p* = 0.014) (Figure S1) [29,30,31]. When analyzing of the overall survival of BCa patients, in 5 of the 20 datasets found (25%), the hazard ratio was superior to 1.15 and in the TCGA-BCa dataset high *TIE2* expression significantly decreased the overall survival of the patients (HR = 1.41, 95% CI = 1.09 to 1.84; *p* = 0.010) (Figure 2B). However, there were more datasets, 9 out of 20 (45%), were the hazard ratio was inferior to 0.85 and a higher expression of *TIE2* is significantly associated to a longer overall survival in 2 datasets: GSE1456 (HR = 0.29, 95% CI = 0.12 to 0.71; *p* = 0.007) and GSE3494 (HR = 0.41, 95% CI = 0.21 to 0.71; *p* = 0.0081) (Figure 2A,B) [29,32].

Together these findings indicate that the receptor TIE2 is of clinical significance in breast cancer and that a high *TIE2* expression could be a marker for a good prognosis as it is associated with a slower progression of the disease, which could be due to induction of dormancy.

### 2.2. Growing Cancer Cells and Primary Breast Tumors Have a Low Expression of TIE2

**Figure 3:**
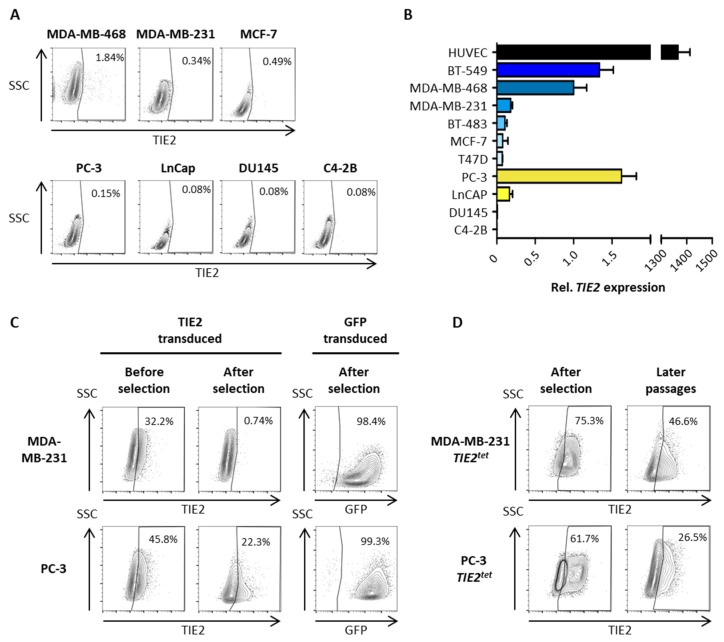
*TIE2* expression is low in cancer cell lines in culture. (**A**) Evaluation of TIE2 protein expression in breast cancer (BCa) and prostate cancer (PCa) cell lines using flow cytometry. (**B**) Evaluation of *TIE2* mRNA expression using RT-qPCR. (**C**) MDA-MB-231 and PC-3 cells were transduced with lentiviral particles to over-express TIE2 or GFP. Expression was measured by flow cytometry five days after transduction (before selection) and three weeks after transduction (after selection). (**D**) MDA-MB-231 and PC-3 cells were transduced to express TIE2 using a Tet-On inducible system. Transduced cells were selected with puromycin, and TIE2 expression was measured by flow cytometry (after selection). MDA-MB-231 cells were then cultured for three weeks, and PC-3 cells were cultured for one week, and TIE2 expression was measured again (later passages). Flow cytometry results are represented as contour plots overlaid with outliers. Values indicate the percentage of TIE2^+^ cells. *TIE2* mRNA expression is represented as the average ± SEM vs. MDA-MB-468 cells.

To further characterize the effect of TIE2 in dormancy and bone metastases, first, we screened different cancer cell lines for the expression of *TIE2*. Using flow cytometry, we tested three different BCa, and four different PCa cell lines to detect the expression of the receptor TIE2 on their membrane. However, we could not detect TIE2^+^ cells among the cell lines tested, except in the BCa cell line MDA-MB-468 (Figure 3A). Although, only a small fraction of these cells (1.84%) were having a detectable expression of TIE2. This absence of TIE2 expression was congruent with mRNA expression levels. Only very low levels of *TIE2* mRNA were detected in the six BCa, and four PCa cell lines tested when compared to the expression in human umbilical cord vein cells (HUVEC) that naturally express *TIE2* (Figure 3B).

Therefore, we decided to constitutively overexpress TIE2 in breast and prostate cancer cells using lentiviral transduction. The coding sequence of the human *TIE2* was cloned into a lentiviral transfer vector, and lentiviral particles were produced. Three days after the transduction of BCa cells MDA-MB-231 and PCa cells PC-3, we detected the expression of TIE2 in 32% and 46% of the cells, respectively (Figure 3C). Despite antibiotic selection with puromycin, there was a sharp decrease in the amount of TIE2^+^ cells, 0.7% and 22%, respectively, in the antibiotic-resistant cells (Figure 3C). A similar result was obtained in independent experiments. In parallel, untransduced cells were all eliminated by puromycin, while after transduction using a GFP-containing lentiviral vector and selection, we obtained more than 97% of GFP^+^ cells (Figure 3C).

Thus, we decided to use a Tet-On inducible expression system to express TIE2 after the antibiotic selection. The coding sequence of *TIE2* was subcloned in the doxycycline (Dox)-inducible transfer vector pCW57.1, and cancer cells were transduced. After puromycin selection and Dox-induction, 75% of MDA-MB-231 and 62% of PC-3 cells were expressing TIE2 (Figure 3D). However, after culturing the cells for several passages, there was also a reduction of the amount TIE2 expressing cells, down to 47% and 27%, in MDA-MB-231 and PC-3 cell lines, respectively, when re-stimulating them with Dox (Figure 3D). Overall, these results indicate that it is not possible to maintain the expression of the TIE2 receptor in proliferating cancer cells in culture.

**Figure 4:**
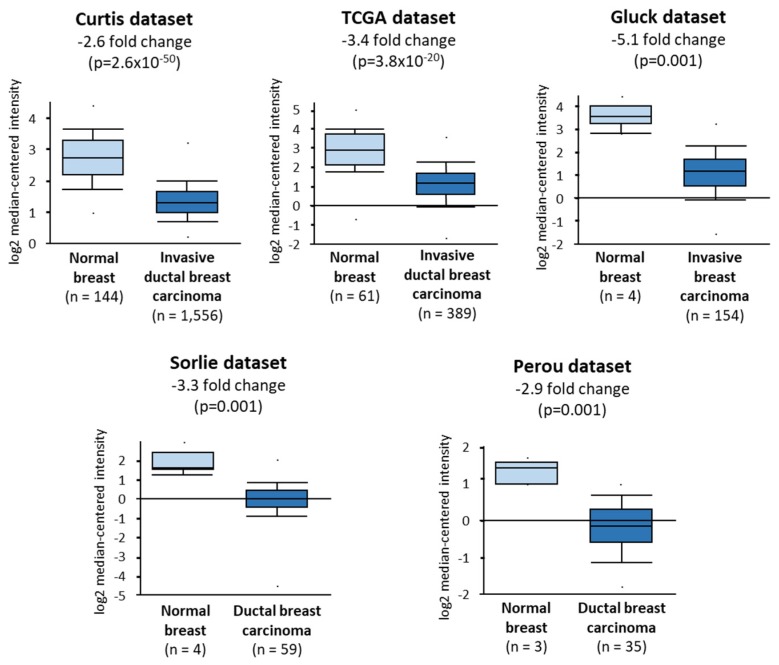
*TIE2* expression is decreased in the primary tumor of breast cancer patients. Relative *TIE2* expression was compared between the normal breast tissue and the primary tumor of BCa patients diagnosed with invasive ductal breast carcinoma [33,34], invasive breast carcinoma [35], or ductal breast carcinoma [36,37]. Relative *TIE2* expressions are represented as box-plots and were compared using an unpaired Student’s *t*-test.

Considering these results, we sought to determine the levels of expression of *TIE2* in the primary tumor of breast cancer patients using the Oncomine database, setting a threshold of 0.001 for the *p*-value and 2 for the fold change. In 5 different independent datasets, we found that the expression of *TIE2* is significantly decreased by more than 2.6-fold, in the primary tumor of breast cancer patients when compared to healthy breast tissue (Figure 4). This result further emphasizes our data and suggest that expression of *TIE2* is lost in proliferating tumor cells in vitro and patients, as it may confer a growth disadvantage, possibly by induction of dormancy.

### 2.3. TIE2 Expression Induces Dormancy of MCF-7 Cells

**Figure 5:**
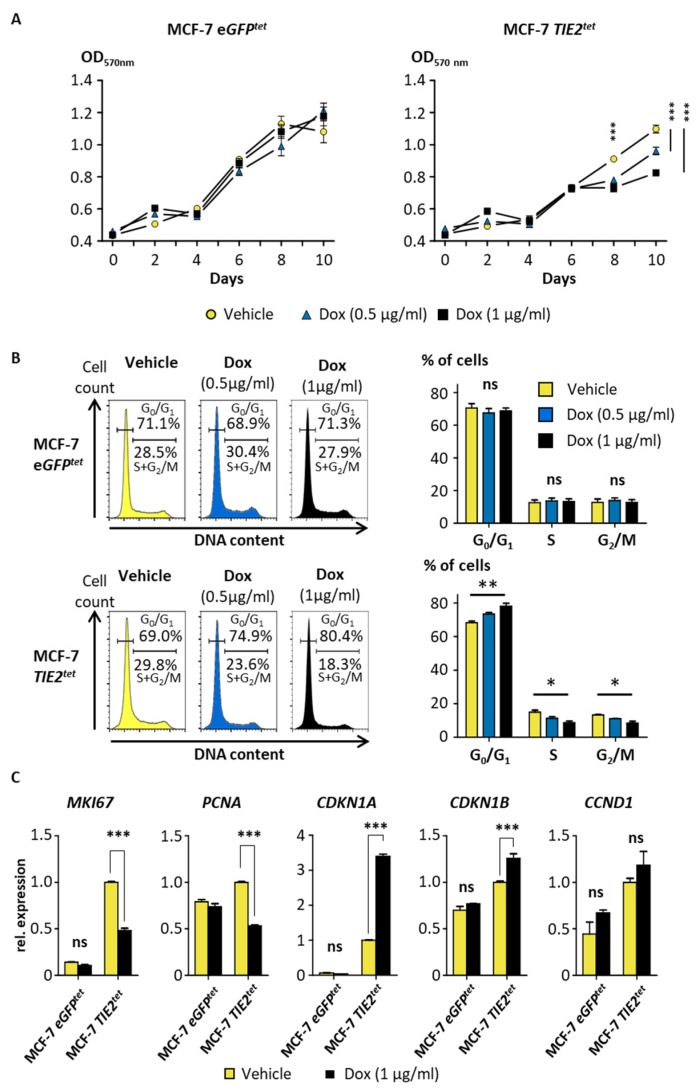
TIE2 expression reduces the proliferation of MCF-7 cells. (**A**) MCF-7 *TIE2^tet^* and MCF-7 *eGFP^tet^* cells were cultured in the presence or absence of doxycycline (Dox, 0.5-1 µg/mL). Proliferation was assessed by MTT. Values are represented as the average ± SEM. *** *p* < 0.001 vs. vehicle-treated cells using a two-way ANOVA with Bonferroni post-test. (**B**) Representative histograms of a cell cycle analysis of MCF-7 *eGFP^tet^* and MCF-7 *TIE2^tet^* cells cultured in the presence or absence of Dox (0.5–1 µg/mL) for 9 days. Percentages of non-proliferating (G_0_/G_1_) and proliferating (S + G_2_/M) cells are indicated. Average percentages of cells in G_0_/G_1_, S, and G_2_/M ± SEM of three independent cell cycle analyses. (**C**) Expression of M*KI67*, *PCNA*, *CDKN1A*, *CDKN1B*, and cyclin D1 (*CCND1*) in *MCF-7 TIE2^tet^* or *eGFP^tet^* cells cultured ±Dox (1 µg/mL) for 9 days. Results are represented as the average gene expression ±SEM vs. MCF-7 *TIE2^tet^* vehicle-treated cells. * *p* < 0.05, ** *p* < 0.01, and *** *p* < 0.001 using a two-way ANOVA with Bonferroni post-test.

To determine whether the expression of the TIE2 receptor induces dormancy, we tested the effect of TIE2 on the proliferation and cell-sensitivity to a chemotherapeutic agent. MCF-7 breast cancer cells were transduced to express TIE2 using the Tet-On system. After puromycin selection and stimulation with Dox, TIE2^+^ MCF-7 were selected by cell sorting. We obtained a population, denominated MCF-7 *TIE2^tet^*, where >85% of the MCF-7 BCa cells expressed TIE2 when cultured for two days in the presence of Dox (Figure S2A). The expression of TIE2 could be maintained without loss for up to 10 days of culture in the presence of Dox (Figure S2B). As a control, we also obtained some MCF-7 *eGFP^tet^* cells that express eGFP in the presence of Dox. Using an MTT assay to assess cell proliferation or viability, we confirmed that Dox or the expression of eGFP did not have any significant effect on the growth of MCF-7 *eGFP^tet^* cells (Figure 5A). However, when MCF-7 *TIE2^tet^* cells were cultured in the presence of Dox, there was a significant, dose-dependent decrease of the amount of viable cells, starting after 8 days of treatment, suggesting that the expression of TIE2 decreased cell proliferation (Figure 5A). To confirm this, cell cycle analyses with Dox treated cells were performed. They showed a significant, dose-dependent decrease in the amount of proliferating MCF-7 *TIE2^tet^* cells (in S and G_2_/M phases) and an increase in the amount of cells in the G_0_/G_1_ phase that can correspond to the induction of dormancy (Figure 5B). In the control cells, MCF-7 *eGFP^tet^*, treatment with Dox did not have any effect on the distribution of the cells in the different phases of the cell cycle (Figure 5B).

Finally, we measured the expression of cell cycle-associated genes using RT-qPCR. A treatment with Dox did not have any effect on the expression of the proliferation marker M*KI67* (or *Ki67*) in control MCF-7 *eGFP^tet^* cells but induced a significant decrease of M*KI67* in MCF-7 *TIE2^tet^* cells (Figure 5C). Similarly, the proliferation marker *PCNA* was significantly down-regulated when TIE2 was expressed in MCF-7 *TIE2^tet^* cells but not when GFP was expressed in the MCF-7 *eGFP^tet^* control cells (Figure 5C) [38]. Accordingly, in MCF-7 *TIE2^tet^* cells, induction of the expression of TIE2 with Dox-induced a significant increase of the cyclin-dependent kinase (CDK) inhibitors *CDKN1A (or P21CIP1)* and *CDKN1B (or P27KIP1)* (Figure 5C), as previously reported in dormant tumor cells [39]. The expression of cyclin D1 (*CCND1*), which is highly expressed in the G_1_ phase before S phase transition, was not significantly up-regulated when MCF-7 *TIE2^tet^* cells expressed TIE2 or when MCF-7 *eGFP^tet^* cells expressed GFP [40].

Together these results indicated that TIE2 expression is sufficient to induce dormancy in MCF-7 breast cancer cells.

### 2.4. TIE2 Reduces Breast Cancer Cell Sensitivity to 5-Fluorouracil

**Figure 6:**
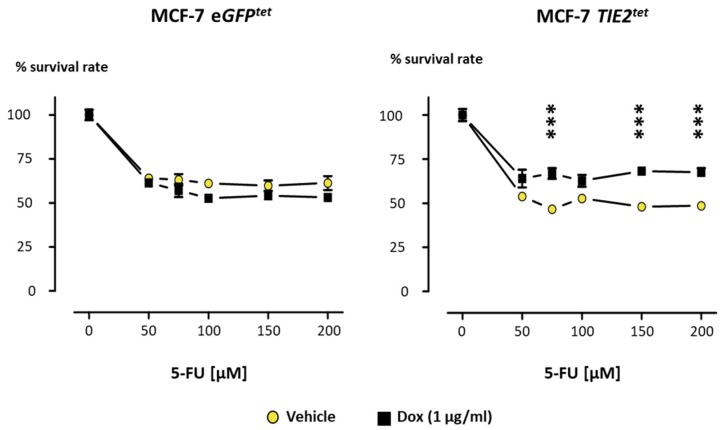
TIE2 expression increases the resistance to 5-Fluorouracil in MCF-7 cells. MCF-7 *TIE2^tet^* and MCF-7 *eGFP^tet^* cells were cultured in the presence or absence of doxycycline (1 µg/mL) during 10 days of the experiment. 5-Fluorouracil (5-FU) was added to the cells for 4 days. Proliferation was assessed by MTT, and values are represented as the average ± SEM. *** *p* < 0.001 vs. vehicle-treated cells and analyzed using a two-way ANOVA with Bonferroni post-test.

Finally, another characteristic of dormant cells is their reduced sensitivity to chemotherapeutic agents due to their lack of proliferation and reduced metabolism [41]. 5-Fluorouracil (5-FU), an analog of the pyrimidine base uracil, is a chemotherapeutic used either as a single drug or in combination with other chemotherapeutics for the treatment of various cancers including BCa [42]. Therefore, we tested whether TIE2 expression and subsequent dormancy could protect MCF-7 cells from 5-FU. MCF-7 *eGFP^tet^* and MCF-7 *TIE2^tet^* cells were cultured in the presence of Dox for 6 days before adding increasing concentrations of 5-FU, and the survival rate was measured 4 days later. 5-FU decreased the amount of viable MCF-7 *eGFP^tet^* cells, and the addition of Dox did not cause any significant changes in the survival rate (Figure 6). The viability of MCF-7 *TIE2^tet^* cells was also decreased by 5-FU. However, Dox-induced expression of TIE2 significantly decreased the effect of 50 to 200 µM 5-FU, resulting in about 40% increase in the survival rate when compared to vehicle-treated MCF-7 *TIE2^tet^* cells (Figure 6). Thus, expression of TIE2 confers to MCF-7 BCa cells a decreased sensitivity to the chemotherapeutic agent 5-FU, which is consistent with the characteristics of dormancy.

### 2.5. TIE2 Expression Reduces Tumor Growth and Bone Metastases In Vivo

**Figure 7:**
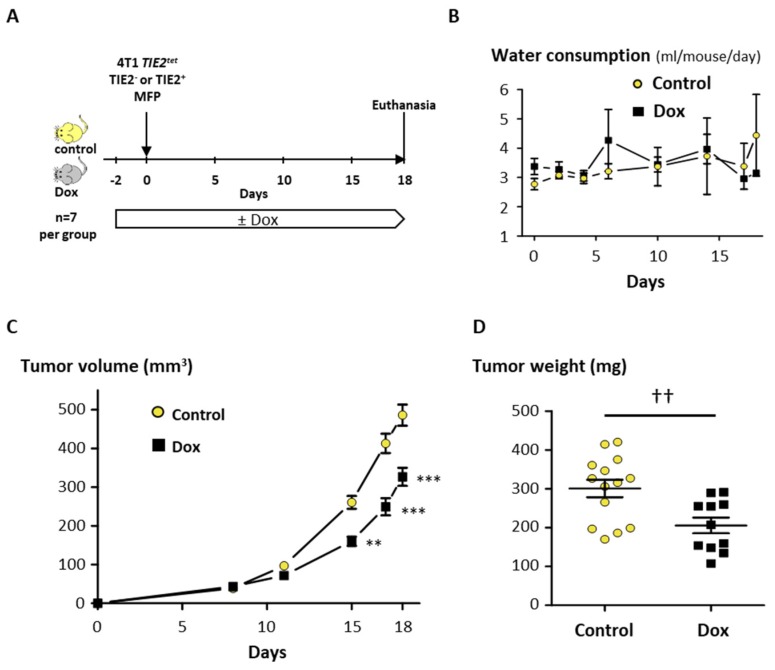
TIE2 reduces the growth of orthotopic 4T1 *TIE2^tet^* tumors. (**A**) 4T1 *TIE2^tet^* cells were cultured in the presence or absence of doxycycline for 2 days before being inoculated bilaterally, in the 4th mammary fat pad of mice receiving or not doxycycline in their drinking water (n = 7 per group). (**B**) Water consumption and (**C**) tumor volume were measured throughout the experiment, and (**D**) the weight of excised tumors was measured at the time of the euthanasia. Results are represented as the average ± SEM. ** *p* < 0.01 and *** *p* < 0.001 vs. control mice using a two-way ANOVA with Bonferroni post-test (panels B and C). ^††^ *p* < 0.01 using an unpaired Student’s *t*-test (panel D).

In order to assess the ability of TIE2 to induce dormancy in vivo, we used the mouse breast cancer cell line 4T1 that can be inoculated in immunocompetent Balb/C mice to induce the formation of mammary fat pad tumors or bone metastases. 4T1 cells were transduced to conditionally express TIE2, and we selected a clone, denominated 4T1 *TIE2^tet^*, that had 99% of TIE2^+^ cells when cultured in the presence of Dox (Figure S2C). We tested first the effect of TIE2 in an orthotopic tumor model. For this, 4T1 *TIE2^tet^* cells, expressing TIE2 or not, were injected bilaterally in the mammary fat pads of Balb/C mice that received or not Dox in their drinking water (Figure 7A). During the experiment, the water consumption was monitored, and there was no difference between the Dox and the control group, indicating that the addition of Dox to the drinking water was well tolerated (Figure 7B). When measuring over time the volume of the tumors, we observed a significant reduction of the tumor growth in the mice receiving Dox, compared to the control group (−33% at day 18, *p* < 0.001) (Figure 7C). As some tumors invaded the peritoneal cavity, at the time of the euthanasia, the tumors were resected and weighed. We found that there was a significant reduction in tumor weight in the mice receiving Dox compared to control mice (−32%, *p* = 0.0056) (Figure 7D). It was described that 4T1 cells could spontaneously metastasize from the mammary fat pad tumor to other organs, including the lungs. However, upon histological examination, we could not detect metastases in the lungs of the mice of either group. To discard the possibility that the decreased tumor growth could be due to Dox, parental 4T1 cells were inoculated in the mammary fat pads, and mice received or not Dox, in a similar manner. We did not observe any reduction of the growth of 4T1 tumors in mice receiving Dox, compared to control mice, and, at the time of euthanasia, there were also no significant differences in the weight of the tumors (Figure S3A,B). These results confirm that the decreased tumor growth measured using 4T1 *TIE2^tet^* cells was not due to Dox but specifically due to TIE2 expression.

**Figure 8:**
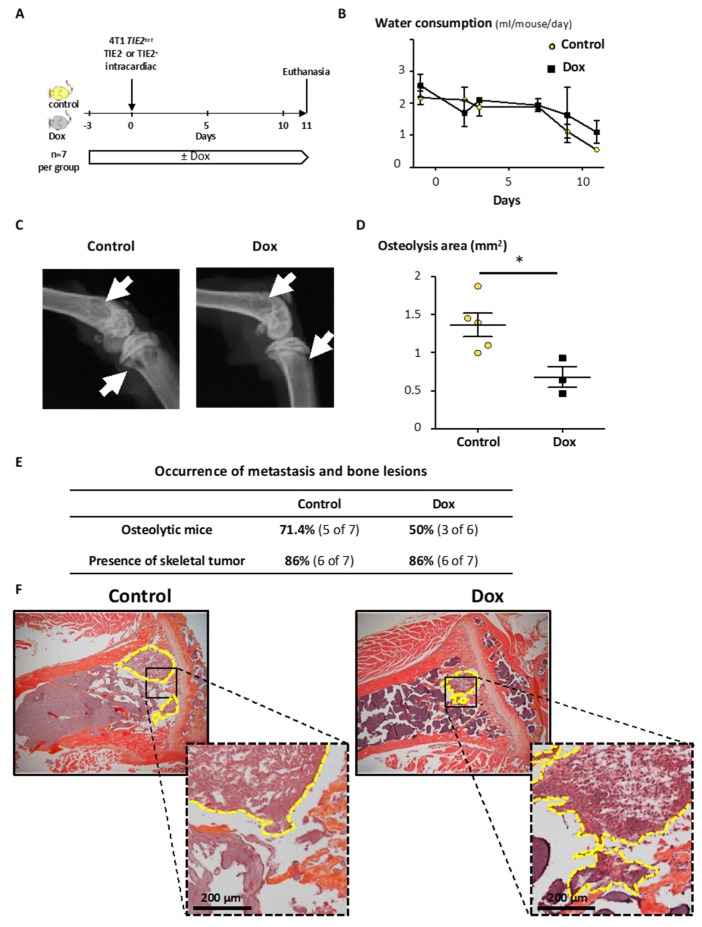
TIE2 reduces osteolysis of 4T1 *TIE2^tet^* bone metastasis. (**A**) 4T1 *TIE2^tet^* cells were cultured in the presence or absence of doxycycline for 2 days before being inoculated in the left cardiac ventricle of mice receiving or not doxycycline in their drinking water (n = 7 per group). (**B**) Water consumption was measured throughout the experiment. Osteolytic lesions were assessed using (**C**) radiographs of the hind limbs of mice (white arrows indicate osteolysis area), and (**D**) the osteolysis area was measured. * *p* < 0.05 using a Mann–Whitney test. (**E**) Summary of the occurrence of osteolytic lesions on endpoint radiographs and of skeletal tumor burden on tissue sections. (**F**) The presence of skeletal tumor burden was assessed using tissue sections stained with hematoxylin, eosin, and orange G (representative sections). The yellow dotted lines contour and indicate the skeletal tumor burden.

To test the effect of TIE2 on the formation of metastases to bone, 4T1 *TIE2^tet^* cells, expressing or not TIE2, were inoculated in the left cardiac ventricle of Balb/C mice. Mice received or not Dox in their drinking water, and as previously, the presence of Dox did not affect their water consumption (Figure 8A). However, starting 9 days after the inoculation of the cancer cells, the mice started drinking less as their condition started to worsen (Figure 8B). As a consequence, it was decided to euthanize the mice. Examination of radiographs of the hind limbs indicated that 71.4% of the control mice presented osteolytic lesions, while 50% of the mice receiving Dox were osteolytic (Figure 8C,E). When measuring the extent of bone lesions, there was a 50% (*p* = 0.024) decrease of the osteolysis area when inducing the expression of TIE2 with Dox compared to the control (Figure 8D). We performed then histological analysis to confirm the presence of cancer cells in the bone marrow cavity of the hind limbs. In both groups, we detected the presence of skeletal tumor burden in 86% of the mice, indicating that TIE2 expression did not prevent or affect the ability of cancer cells to disseminate to the bone marrow (Figure 8E,F). To also exclude that Dox has an effect on the osteolytic capability of 4T1 cells, we inoculated parental 4T1 cells in the left cardiac ventricle of Balb/C mice. Mice received or not Dox in their drinking water, and bones were collected ten days later. We assessed the osteolytic lesions on radiographs and did not find any difference in the occurrence (82% in the control group vs. 80% in the Dox group) or extent of the osteolysis (*p* = 0.73) (Figure S3C,D).

Overall, these results show that, although it did not affect the capacity of 4T1 *TIE2^tet^* cells to home to the bone, the induction of the expression of TIE2 decreased the development of osteolytic bone metastases in mice, as well as the growth of the primary tumor.

## 3. Discussion

During this study, we hypothesized that the expression of the receptor TIE2 regulates the dormancy of BCa cells, causing increased resistance to chemotherapeutic agents and reducing the development of osteolytic metastases.

Cancer cells can disseminate from the primary tumor to sites of metastases, such as the bone marrow, during the initial stages of cancer. Up to 25% and 33% of patients with stage pT1 and pT2 BCa, respectively, were found to have DTCs in bones [43]. Despite their presence in bone, DTCs will not immediately form bone metastases when they enter a phase called dormancy [5]. During this stage, their metabolism decreases, and their cell cycle is paused in the G_0_ phase. Due to the lack of proliferation, dormant cancer cells are resistant to radio- or chemotherapy. As a consequence, dormant cells can persist as residual disease in treated patients and cause a recurrence in the form of bone metastases sometimes after decades of life apparently free of cancer [3]. DTCs and HSCs share similarities in the way they are retained within the bone marrow and are rendered dormant, which allowed the identification of some dormancy-inducing factors, such as BMP7 or GAS6 [44]. However, it has not been possible to implement them in clinical trials so far. Angiopoietin-1 and its receptor TIE2 cause the dormancy and persistence of HSCs in the bone marrow [19]. As such, TIE2 may also cause DTCs to enter dormancy in the bone marrow. Since there already exist some agents that inhibit the Angiopoietin-TIE2 axis and that are being tested in patients with cancer as inhibitors of angiogenesis, TIE2 could be a more reachable target for the treatment of dormant DTCs. Thus, the aim of our study was to characterize the role of TIE2 in the dormancy of breast cancer bone metastases and the progression of the disease, using a combination of in vitro experiments, pre-clinical models, and patient data.

First, we screened ten different established BCa and PCa cell lines for the expression of *TIE2* but could not find detectable levels of the protein and extremely low levels of mRNA (>1300 times less than in HUVEC) in cultured cells. This observation is quite similar to Tang et al. who found that less than 0.4% of PCa cells of the tested cell lines expressed TIE2 protein in culture [20]. Interestingly, *TIE2* expression was very low or absent regardless of the cell type tested here (BCa vs. PCa), the expression of the estrogen or androgen receptor, or the induction of osteolytic or osteoblastic lesions. To test the effect of TIE2 on BCa cells, we managed to modify and select by cell-sorting some MCF-7 cells that conditionally express TIE2 in the presence of Dox. The expression of TIE2 in MCF-7 specifically induced a decrease in cell proliferation and an increase in the number of cells in the G_0_/G_1_ phase. These results correlate with a decrease in the expression of the proliferation markers *MKI67* and *PCNA* when MCF-7 *TIE2^tet^* expressed TIE2, as well as an increase of the markers of cell cycle arrest *CDKN1A* and *CDKN1B*, already found to be increased during BMP7-induced dormancy in PCa cells [45]. Since the expression of TIE2 in MCF-7 also decreased their sensitivity to 5-FU, these results concur to indicate that the expression of TIE2 induces the dormancy of MCF-7 BCa cells, in vitro. Further studies would be required to determine the molecular mechanisms and signaling pathways through which the receptor TIE2 regulates dormancy in BCa cells.

To confirm that TIE2 can also control dormancy in vivo, we used 4T1 cells, a triple-negative BCa cell line derived from a Balb/C mouse that can be used as a syngeneic model of BCa and bone metastases. Upon inoculation in mice, the expression of TIE2 caused a significant decrease of the growth of orthotopic tumors, as well as of the development of osteolytic metastases. In contrast, the development of orthotopic tumors and osteolytic metastases of parental 4T1 was not affected by Dox.

Overall, these results demonstrate that TIE2 alone is sufficient to induce the dormancy of BCa in vitro and in vivo in mice. This is similar to the dormancy induced in PCa cells PC-3 in vitro, while in vivo expression of TIE2 seemed to increase the homing of PC-3 cells to bone (25% of mice with bone metastases for PC-3 TIE2^High^ vs. 0% of TIE2^Low^ cells) [20]. In our model, the expression of TIE2 did not affect the capacity of 4T1 cells to disseminate to the bone as 86% of the inoculated mice had cancer cells in the bone marrow regardless of TIE2 expression. If TIE2 did not change the homing of BCa cells to bone, it was, however, able to decrease the occurrence and development of osteolysis in mice.

These in vitro and in vivo results are consistent with the effect of *TIE2* expression on the progression and the outcome of the disease in BCa patients. Indeed, we found in six different datasets from the PROGgene database that higher levels of *TIE2* mRNA in the primary tumor is associated with a significantly longer overall survival, relapse-free survival, or metastasis-free survival. Despite that, there were 7 independent datasets where higher levels of *TIE2* were associated with a decreased overall or relapse-free survival (HR >1.15). Unfortunately, we could not determine whether this was due to differences in the composition of the cohorts of patients (tumor stage, prior treatments, ER, HER2, or PR status), which would need to be further explored. However, the hazard ratio was lower than 0.85 in 22 different, independent datasets of patients, indicating that overall BCa patients with high *TIE2* expression are less likely to have a relapse, metastases, or a fatal outcome. This increased time before the progression of the disease is consistent with an increased duration of the dormancy phase due to high *TIE2* levels. In a study on oral squamous cell carcinoma (OSCC), expression of TIE2 was associated with a decrease of the migration and invasion of OSCC cells in vitro. In the clinic, none of the patients with a high immunohistochemistry score for TIE2 (0 of 18) had developed regional lymph node metastases, compared to 33% of the patients with a low TIE2 score (17 of 52, *p* = 0.008), which is consistent with our findings [46]. Han et al. have shown that HUVECs induced a dormancy-like effect in MCF-7 cells in vitro, in the presence of ANGPT1 [47]. However, as MCF-7 cells do not express detectable levels of TIE2, it stands to reason that the observed effect was due to factors released by the HUVECs, like Thrombospondin-1, as stated by the authors, rather than the activation of TIE2 in MCF-7 cells [47]. However, in our models, it is the expression of TIE2 in the BCa cells that regulates their behavior. Therefore, it is the first time that it is reported that TIE2 in BCa cells induces dormancy, delays bone metastases in mice, and is a good-prognosis factor in patients.

Since the expression of *TIE2* is inhibiting the proliferation of cancer cells by driving dormancy, it explains why *TIE2* is not detected, or only at very low levels, in breast as well as prostate cancer cells proliferating in culture. *TIE2* expression would confer a growth disadvantage to these cells that will be outgrown by cancer cells with lower expression. This is consistent with our observation that the expression of *TIE2* is decreased in biopsies of the primary tumor of patients with invasive BCa when compared to samples of normal breast tissues. Since these samples are likely to contain non-cancerous cells in the tumor or non-epithelial cells the normal tissue, we need to be cautious in the interpretation that *TIE2* expression is changed in cancer cells compared to normal mammary epithelial cells. In vitro, it would be interesting to compare the expression of TIE2 between cancer cell lines and immortalized normal cells. However, another study also found, in a similar manner, that the expression of TIE2 was very low in different cell lines of OSCC, and decreased in the primary tumor of patients with OSCC when compared to normal oral epithelia [46]. If the expression of TIE2 is lost or reduced in the primary tumor, it seems, however, that it can be restored or increased at the site of bone metastases. Werbeck et al. compared gene expression at different sites of tumors caused by Met-1 cells, derived from the primary tumor of a MMTV-PyMT mouse [48]. Levels of *TIE2* mRNA were higher in Met-1 cells in the lungs and the adrenal glands, and significantly higher in cells growing in the tibia (3.8-fold, *p* = 0.03), compared to cells inoculated in the mammary fat pad [48]. Interestingly, the proliferation index of Met-1 cells was lower in bone than in mammary fat pad tumors. This, according to our results, could be related to the higher *TIE2* expression causing dormancy. Changes in the levels of *TIE2* suggest that the expression of the gene is likely to be under the influence of local factors. DTCs colonizing bone are known to settle in the osteoblastic HSC niche where they fall under the influence of the proteins produced in this particular microenvironment, or its physicochemical conditions [12,13,49]. Since the osteoblastic HSC niche is located at the end of an oxygen gradient, it is hypoxic, which helps to maintain the dormancy and stemness of HSCs. Hypoxia also increases the expression of *TIE2* in endothelial cells, which correlates well with the observation that it is only the HSCs in the hypoxic osteoblastic niche that are TIE2^+^, while HSCs in the endothelial niche did not retain TIE2 expression [19,50]. In addition, osteoblasts in the HSC niche are the primary producer of ANGPT1 [19]. Therefore, the TIE2 receptor may be expressed in the DTCs after their arrival in the hypoxic HSC niche, and subsequently activated by osteoblast-derived ANGPT1 to induce dormancy. Although the sole expression of TIE2 is enough to induce dormancy in BCa in vitro and to decrease tumor growth or bone metastases in mice, it is not entirely abrogating cell proliferation or tumor growth. Therefore, it is likely that dormancy lasting for years to decades in patients is a multifactorial phenomenon, driven by a combination of other additional factors, such as BMP7, GAS6, or JAG1 [44].

It is still unclear why DTCs escape from this dormancy phase, and it remains to be determined why TIE2-induced dormancy gets interrupted, causing a relapse in the form of bone metastases. However, it is possible that anti-TIE2 treatments cause such a side-effect. Due to the role of TIE2 and ANGPT1 in tumor-associated angiogenesis, TIE2 is a target for anti-angiogenic therapies, and anti-TIE2 agents have been developed. AMG-386 is a small peptibody against the TIE2 ligands ANGPT1 and ANGPT2, and its efficacy is currently tested against different types of cancer, including BCa, in phase 1 and 2, clinical trials (NCT00511459, NCT00807859, and NCT01042379) [51]. Similarly, Rebastinib, a small molecule inhibitor of the kinase domain of TIE2, but also of the Abelson tyrosine-protein kinase proto-oncogene 1 (ABL1) can be used to inhibit angiogenesis and is tested for the treatment of BCa patients in clinical trials of phase 1 and 2 (NCT02824575, NCT03717415, and NCT03601897) [23,52]. In addition to their beneficial anti-angiogenic effects, these compounds could reverse the dormancy inducing effect of TIE2 in DTCs in the bone marrow, and put patients at risk for bone metastases. Since cells that did not express TIE2 were more sensitive to the chemotherapeutic agent 5-FU, it is possible though that anti-TIE2 agents have the same effect and that their combination with chemotherapy will permit the elimination of awakened DTCs as they resume their proliferation. In a pre-clinical model, a combination of Rebastinib with the microtubule inhibitor Paclitaxel was more efficient at decreasing tumor growth in the mammary fat pad, as well as the development of lung metastases [23]. In five of the six clinical trials testing anti-TIE2 therapy in BCa patients, the treatment with Rebastinib or AMG-386 is combined with a chemotherapeutic agent, such as Paclitaxel, Eribulin, Capecitabine, or Carboplatin that could protect patients from undesired effects on dormant DTCs.

## 4. Materials and Methods

### 4.1. Plasmids and Subcloning

For the over-expression of eGFP or human TIE2 in cancer cells, we used a third-generation lentiviral system consisting of the packaging vectors pLP1 and pLP2 (Thermo Fisher Scientific, Waltham, MA, USA), the envelope vector pMD2.G (a gift from Didier Trono (Addgene plasmid # 12259)) and the transfer vectors pLJM1-eGFP (a gift from David Sabatini (Addgene plasmid #13319)) [53] or pCW-Cas9 (a gift from Eric Lander and David Sabatini (Addgene plasmid #50661)) [54]. For the constitutive over-expression of TIE2, we used a pLJM1 vector with a cytomegalovirus (CMV) promoter. The ORF of the human *TIE2* sequence was amplified using the Q5 High-Fidelity DNA Polymerase (New England Biolabs, Ipswich, MA, USA) and the IMAGE clone 5228999 (PlasmID Repository, Harvard Medical School, Boston, MA, USA) with the oligonucleotides 5′-TTAGTGAACCGTCAGATCCGCTAGCATGGACTCTTTAGCCAGCTTAG-3′ and 5′-CCATTTGTCTCGAGGTCGAGAATTCCTAGGCCGCTTCTTCAGCAGA-3′. The amplification product was inserted in the pLJM1-eGFP vector linearized with NheI and EcoRI enzymes (New England Biolabs, Ipswich, MA, USA) using the Gibson Assembly Cloning kit (New England Biolabs, Ipswich, MA, USA) to obtain the pLJM1-hTIE2 plasmid. For conditional expression, we used a Tet-ON system with the pCW57.1 backbone. The coding sequences of *eGFP* and *hTIE2* were amplified from the pLJM1-eGFP and pLJM1-hTIE2 plasmid by PCR, using the oligonucleotides 5′-CAGATCGCCTGGAGAATTGGAACCGTCAGATCCGCTAGC-3′ and 5′-TACCGTCGACTGCAGAATTCTATTTGTCTCGAGGTCGAGAATTC-3′. The PCR products were inserted in the pCW-Cas9 vector linearized with NheI and BamHI enzymes (New England Biolabs, Ipswich, MA, USA), using the Gibson Assembly Cloning kit to obtain the pCW-eGFP and pCW-hTIE2 plasmids.

### 4.2. Cell Culture and Transfection

Breast cancer cell lines BT-549, BT-483, MDA-MB-231, MDA-MB-468, MCF-7, and T47D, and prostate cancer cell lines PC-3, LnCAP, DU145, and C4-2B as well as human umbilical cord vein endothelial cells (HUVEC) and human embryonic kidney cells HEK-293T were obtained from the American Type Culture Collection (ATCC, Manassas, VA, USA). HEK-293T, MDA-MB-231, and MCF-7 cells were grown in high-glucose DMEM medium (Corning, Corning, NY, USA), MDA-MB-468 in L-15 medium (Corning, Corning, NY, USA), T47D, PC-3, LnCAP, DU145, and C4-2B in RPMI medium (Corning, Corning, NY, USA), and BT-483 and BT-549 cells in RPMI medium (Corning, Corning, NY, USA) containing 10 µg/mL and 0.8 µg/mL insulin, respectively. All media were supplemented with 10% fetal bovine serum (Biowest, Riverside, MO, USA), 100 unit/mL penicillin, 100 μg/mL streptomycin, and 250 ng/mL Amphotericin B (Corning, Corning, NY, USA). HUVEC cells were grown in Vascular Cell Basal Medium (ATCC, Manassas, VA, USA) supplemented with the Endothelial Cell Growth Kit-VEGF (ATCC, Manassas, VA, USA) and complemented with 10 U/mL Penicillin and 10 µg/mL Streptomycin (Corning, Corning, NY, USA). All cells were grown at 37 °C, in a humidified atmosphere with 5% CO_2_.

To assess TIE2 expression, HUVECs and cancer cells were stained with a fluorescently labeled antibody against TIE2 (clone 33.1, BioLegend, San Diego, CA, USA) and analyzed on an Attune^®^ Acoustic Focusing Flow Cytometer (Thermo Fisher Scientific, Waltham, MA, USA). Singlets were gated using FSC-area vs. FSC-height, and SSC-area vs. SSC-height density plots and cells were gated using FSC-area vs. SSC-area density plots. A minimum of 10,000 cells was recorded and analyzed.

Lentiviral particles were generated in 293T cells co-transfected with equimolar amounts of pMDG.2, pLP1, pLP2, and the transfer vector pLJM1 or pCW, using Lipofectamine 2000 (Thermo Fisher Scientific, Waltham, MA, USA), according to the manufacturer’s instructions. Lentiviral particles were harvested 48 and 72 h after transfection and mixed with polybrene (8 µg/mL, Sigma, Saint-Louis, MO, USA) before transduction. Protein expression was confirmed by flow cytometry 3 days after the transduction. Transduced cells were selected using 0.5 to 4 µg/mL of puromycin, depending on the cell line transduced.

MCF-7 *TIE2^tet^* cells with inducible expression of TIE2 were cultured in doxycycline (Dox, 1 µg/mL, 48 h) (Sigma, Saint-Louis, MO, USA), stained with an anti-TIE2 antibody, and selected using a MoFlo XDP cell sorter (Beckman Coulter, Brea, CA, USA). For 4T1 *TIE2^tet^* cells, a monoclonal cell line was generated from a polyclonal pool of transduced cells using limiting dilution.

### 4.3. Cell Proliferation and Cell Cycle Analysis

Parental MCF-7, MCF-7 e*GFP^tet^*, and MCF-7 *TIE2^tet^* cells were seeded in 96-well plates or 6 cm dishes (12,500 cells per cm^2^) and grown for 48 h before culturing them or not in the presence of Doxycycline (Dox, 0.5–1.0 µg/mL) (Sigma, Saint-Louis, MO, USA) for up to 10 days before assessing cell proliferation or cell cycle analysis. Due to the short half-life of Dox in the culture medium (24 h), Dox was replenished every 24 h. The culture medium was changed every 72 h. In some experiments, 50 to 400 µg/mL of 5-Fluorouracil (BioBasic, Markham, ON, Canada) was added to the media after six days of treatment with Dox.

Cell proliferation assay was done as described previously [55]. Briefly, MTT (Sigma, Saint-Louis, MO, USA) was added to a final concentration of 833.3 µg/mL. Cells were incubated for 4 h, lysed with an SDS (10%) solution in HCl (10 mM), and stored at 37 °C overnight. Absorbance was measured at 570 nm on a spectrophotometer (Epoch, BioTek, Winooski, VT, USA). In experiments where cells were treated with 5-FU, the survival rate measured by calculating the ratio of the OD570 nm of cells treated with 5-FU and the OD570 nm of cells treated with the vehicle DMSO.

For cell cycle analysis, cells were trypsinized after 9 days of Dox treatment. Two million cells were fixed overnight in ethanol (70%) at −20 °C, and then washed with PBS. Pelleted cells were then re-suspended in PBS with 5% FBS (Corning, Corning, NY, USA), Propidium Iodide (50 µg/mL) (Sigma, Saint-Louis, MO, USA), and RNAse A (50 µg/mL) (Sigma, Saint-Louis, MO, USA), and incubated in the dark at room temperature for 30 min. Flow cytometry analysis was performed on an Attune^®^ Acoustic Focusing Flow Cytometer (Thermo Fisher Scientific, Waltham, MA, USA). Singlets were plotted by height and area of Propidium Iodide signal intensity. A minimum of 50,000 single cells was recorded and analyzed using FlowJo (v10.6.1, Becton, Dickinson and Company, Franklin Lakes, NJ, USA).

### 4.4. Gene Expression Analysis

Total RNA was extracted from cells using the GenElute™ Mammalian Total RNA Miniprep Kit (Sigma, Saint-Louis, MO, USA) or the GeneJET RNA Purification Kit (Thermo Fisher Scientific, Waltham, MA, USA) according to the manufacturer’s protocol. RNA (125 ng to 400 ng) was reverse-transcribed using anchored oligo dT primers (Thermo Fisher Scientific, Waltham, MA, USA), and SuperScript™ II reverse transcriptase (Thermo Fisher Scientific, Waltham, MA, USA) according to vendor’s instructions. cDNAs were analyzed in triplicate by quantitative real-time PCR using 7500 Real-Time PCR System (Thermo Fisher Scientific, Waltham, MA, USA) with SYBR™ Green PCR Master Mix (Thermo Fisher Scientific, Waltham, MA, USA) or QuantiTect SYBR^®^ Green Master Mix (Qiagen, Hilden, Germany). The oligonucleotides used (T4Oligo, Irapuato, México) are listed in Table 1. Gene expression was normalized by using *RPL32* as housekeeping gene.

### 4.5. Animal Studies

All animal experiments were performed in compliance with the guidelines of the Mexican Official Standard (NOM-062-ZOO-1999, Especificaciones técnicas para la producción, cuidado y uso de los animals de laboratorio), and were approved by the institutional ethics committee of the CICESE (ethical reference number: ANIM_TERR_2020_01). Balb/C mice (Balb/cAnNHsd) obtained from Harlan-Envigo (Indianapolis, IN, USA), or subsequently bred a the CICESE were maintained in an Optimice cage system (Animal Care Systems, Centennial, CO, USA), in a controlled environment (24 °C and 12 h light/dark cycle), and provided ad libitum with water and food (2018S Teklad Global 18% protein rodent diet, Envigo, Indianapolis, IN, USA). Mice were acclimated for at least a week before starting the experiments.

For orthotopic tumors, 5- to 6-week-old Balb/C mice started receiving drinking water with sucrose (0.5%) supplemented or not with Dox (0.5 mg/mL) [56]. Two days later, mice were injected with parental 4T1 cells or 4T1 *TIE2^tet^* cells, bilaterally, in the 4th mammary fat pads (10^5^ cells in 50 µL PBS). Mouse weight and food and water consumption were monitored. Tumor growth was monitored over 18 days, using a Vernier caliper, and the tumor volume was calculated using the formula: Volume = [length × (width)^2^]/2. After euthanasia using pentobarbital overdose (630 mg/kg) (PiSA Farmaceutica, Guadalajara, México), followed by cervical dislocation. Tumors were excised and weighted.

For bone metastasis experiment, 5-6-week-old Balb/C mice received or not Dox (0.5 mg/mL) in their drinking water. Three days later, anesthetized mice were injected with parental 4T1 cells or 4T1 *TIE2^tet^* cells (10^5^ cells in 100 µL PBS) in the left cardiac ventricle. Eleven days later, mice were euthanized, and hind limb bones were collected, fixed in buffered formalin, and stored in ethanol (70%). Radiographs were taken using an InVivo XTreme (Bruker, Billerica, MA, USA) at the Laboratorio Nacional de Microscopia Avanzada (Instituto de Biotecnología, UNAM, Cuernavaca, México). The area of osteolysis was identified as radiolucent lesions and measured manually using ImageJ. Bones were then decalcified using EDTA solution (0.34 M), during three weeks, processed in an STP120 tissue processor (Thermo Fisher Scientific, Waltham, MA, USA), and embedded in paraffin for sectioning. Sections (7 µm thickness) were cut using an HM 340E Electronic Rotary Microtome (Thermo Fisher Scientific, Waltham, MA, USA) and stained with hematoxylin and eosin (H&E). Images were collected using a Zeiss Axio Scope A1 microscope (Zeiss, Oberkochen, Germany) with an Axiocam 505 color (Zeiss, Oberkochen, Germany).

### 4.6. Data Mining

To evaluate the prognostic value of *TIE2* mRNA in the primary tumor of patients with breast cancer, the PROGgene V2 database was used [24]. Relapse-free, metastasis-free, or overall survival was compared between high and low expression groups using the median gene expression value as the bifurcating point. Statistics were calculated by the web-tool using a log-rank test.

Expression of *TIE2* mRNA in breast cancer and normal breast tissue samples of patients was queried using the Oncomine database. The *p*-values presented were extracted directly from the Oncomine analysis, and the tests have not been repeated manually.

### 4.7. Statistical Analysis

Statistical analyses were performed using the GraphPad Prism software v5.0 (GraphPad Software, Inc., San Diego, CA, USA). Groups of two were first analyzed with a D’Agostino and Pearson omnibus normality test, and a Shapiro–Wilk normality test and then either a Student’s *t*-test (Gaussian distribution) or a Mann–Whitney *t*-test (non-Gaussian distribution or small n) was performed. Comparisons of three or more groups were conducted using a 1-way ANOVA test, followed by a Bonferroni’s post-test. For responses that were affected by two variables, a 2-way ANOVA with a Bonferroni’s post-test was used. Results are expressed as the mean ± SEM, and a *p* ≤ 0.05 was considered significant.

## 5. Conclusions

In this work, we showed that the tyrosine kinase receptor TIE2 is able and sufficient to induce dormancy in BCa cells in vitro and to reduce tumor growth and bone metastasis in vivo. In BCa patients, a higher expression of *TIE2* is associated with an increased time until the development of metastases and relapse as well as an increased survival that can be explained by a prolonged dormancy. Considering that inhibiting TIE2 could re-awaken dormant TIE2^+^ DTCs in the bone marrow, we should consider associating chemotherapies to the treatment of patients that undergo novel anti-TIE2 therapies, to reduce the risk of bone metastases.

## Figures and Tables

**Table 1:** Sequences of oligonucleotides used for real-time RT-qPCR.

Gene	Gene ID	Orientation	Sequence
*TIE2*	7010	forward	TACACCTGCCTCATGCTCAG
		reverse	TTCACAAGCCTTCTCACACG
*CDKN1A*	1026	forward	ATGAAATTCACCCCCTTTCC
		reverse	CCCTAGGCTGTGCTCACTTC
*CDKN1B*	1027	forward	CAGGTAGTTTGGGGCAAAAA
		reverse	ACAGCCCGAAGTGAAAAGAA
*MKI67*	4288	forward	AAGCCCTCCAGCTCCTAGTC
		reverse	GCAGGTTGCCACTCTTTCTC
*PCNA*	5111	forward	TCTGAGGGCTTCGACACCTA
		reverse	TCTCCTGGTTTGGTGCTTCA
*CCND1*	1029	forward	ATCAAGTGTGACCCGGACTG
		reverse	CTTGGGGTCCATGTTCTGCT
*RPL32*	6161	forward	CAGGGTTCGTAGAAGATTCAAGGG
		reverse	CTTGGAGGAAACATTGTGAGCGATC

## Supplementary Materials

The following are available online at https://www.mdpi.com/2072-6694/12/4/868/s1, Figure S1: High expression of *TIE2* in the primary tumor of breast cancer patients is associated with an increased time to the relapse, Figure S2: Selection of MCF-7 *TIE2^tet^* and 4T1 *TIE2^tet^* cells with inducible expression of TIE2, Figure S3: Doxycycline does not affect the growth of mammary fat pad tumors growth or the formation of osteolysis in mice.

## Abbreviations

5-FU	5-fluorouracil
ANOVA	analysis of variance
BCa	breast cancer
Dox	doxycycline
DTC	disseminated tumor cell
eGFP	enhanced green fluorescent protein
HSC	hematopoietic stem cell
HUVEC	human umbilical vein endothelial cell
MTT	Methylthiazolyldiphenyl-tetrazolium bromide
OSCC	oral squamous cell carcinoma
PCa	prostate cancer
SEM	standard error of the mean
Tet	tetracycline
