# Supplementary material for: TIE2 Induces Breast Cancer Cell Dormancy and Inhibits the Development of Osteolytic Bone Metastases
Source: Cancers (Basel). 2020 Apr 3;12(4):868. doi: 10.3390/cancers12040868 (PMC7226250; doi:10.3390/cancers12040868)
Supplement: Supplementary file 1 [file cancers-12-00868-s001.pdf]

Supplementary Materials: The Receptor TIE2 Induces Breast Cancer Cell Dormancy and Inhibits the Development of Osteolytic Bone Metastases

Florian Drescher, Patricia Juárez, Danna L. Arellano, Nicolás Serafín-Higuera, Felipe Olvera-Rodriguez, Samanta Jiménez, Alexei F. Licea-Navarro and Pierrick GJ. Fournier

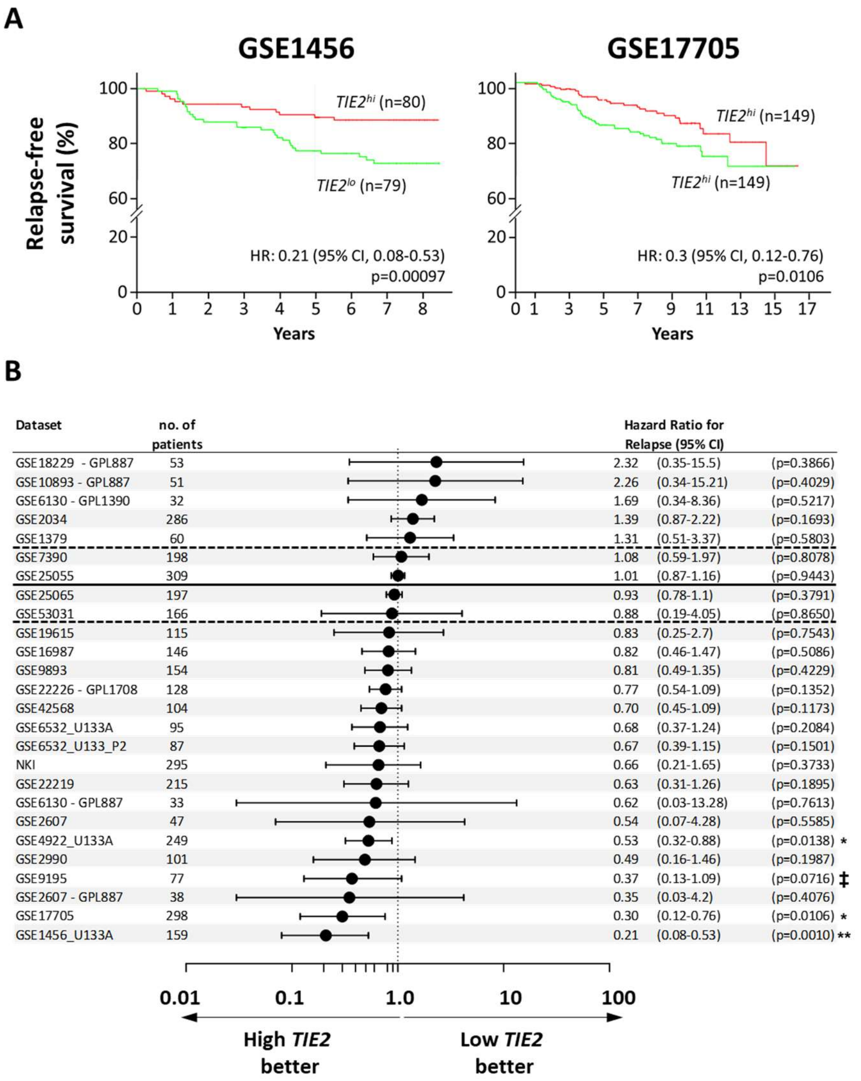

Figure S1. High expression of *TIE2* in the primary tumor of breast cancer patients is associated with an increased time to relapse. Analysis of relapse-free survival using the PROGgene database. The median *TIE2* mRNA level in the primary tumor was taken as a bifurcation point. Results are presented as (**A**) Kaplan-Meyer plots for the Pawitan (GSE1456) and Symmans datasets (GSE17705), or as (**B**) a forest plot, indicating the overall hazard ratio (HR) for metastasis-occurrence and 95% confidence interval (CI). The horizontal line divides datasets with a HR higher or lower than 1, and the dotted lines separate the datasets with a HR lower than 0.85 and higher than 1.15. Survival analysis was performed using a log-rank test, ‡ $p < 0.078$, * $p < 0.05$, and ** $p < 0.01$.

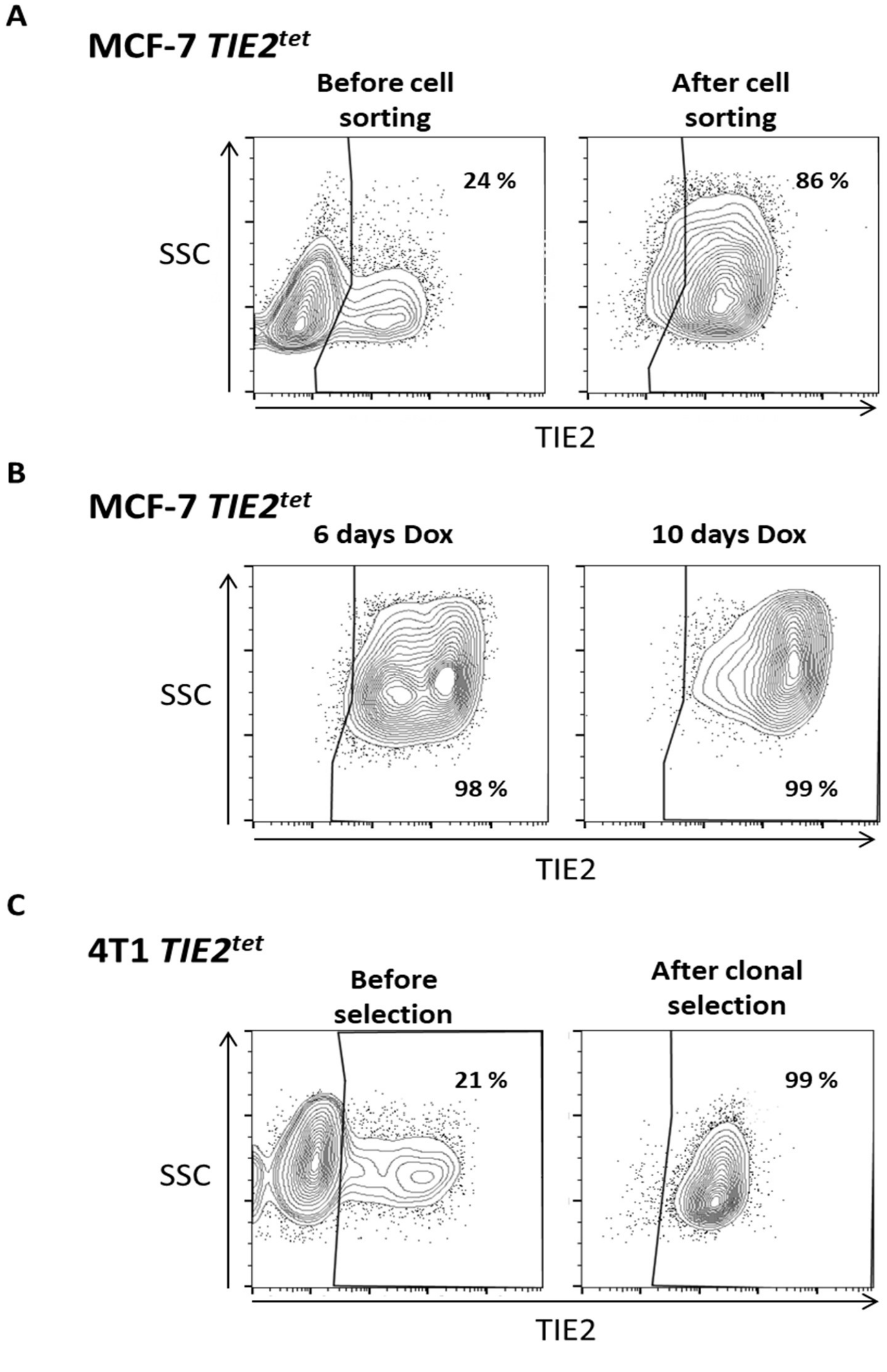

Figure S2. Selection of MCF-7 *TIE2*[tet] and 4T1 *TIE2*[tet] cells with inducible expression of TIE2. (**A**) TIE2 expression was induced in MCF-7 *TIE2*[tet] with Dox (1 µg/mL, 48 h) before isolating TIE2[+] cells using a cell sorter. After expansion, TIE2 expression of sorted cells was evaluated by flow cytometry after 48 h of culture in Dox (1 µg/mL). (**B**) To confirm the stability of TIE2 expression during the proliferation assays, TIE2 expression was assessed by flow cytometry after culturing the sorted cells for up to ten days in the presence of Dox (1 µg/mL). (**C**) A monoclonal cell line was selected from a polyclonal pool of 4T1 *TIE2*[tet] cells (before selection) using limiting dilution. Using flow cytometry, we confirmed that 99% of the cells are TIE2[+] in the monoclonal 4T1 *TIE2*[tet] cell line (after clonal selection). Flow cytometry results are represented as contour plots overlaid with outliers.

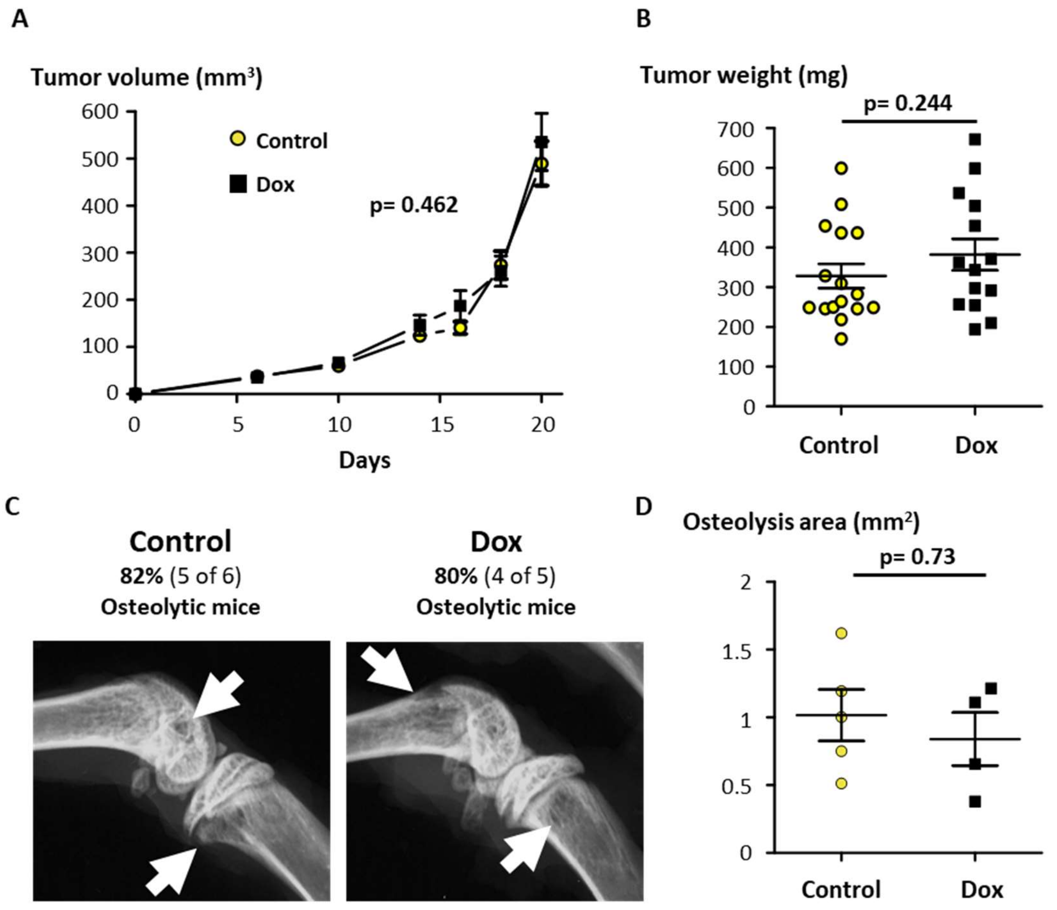

Figure S3. Doxycycline does not affect the growth of mammary fat pad tumors growth or the formation of osteolysis in mice. Parental 4T1 cells were inoculated bilaterally, in the 4th mammary fat pad of mice receiving or not doxycycline in their drinking water ($n = 8$ per group). (**A**) Tumor volumes were measured throughout the experiment, and (**B**) the weight of excised tumors was measured at the time of the euthanasia. Parental 4T1 cells were inoculated in the left cardiac ventricle of mice receiving or not doxycycline in their drinking water. Osteolytic lesions were assessed using (**C**) radiographs of the hindlimbs of mice (white arrows indicate osteolysis area), and (**D**) the osteolysis area was measured. Results are represented as the average ± SEM. Tumor volumes were compared using a two-way ANOVA (**A**), and tumor weight and osteolysis area were compared using a Mann-Whitney's *U* test (**B**, **D**).

© 2020 by the authors. Licensee MDPI, Basel, Switzerland. This article is an open access article distributed under the terms and conditions of the Creative Commons Attribution (CC BY) license (http://creativecommons.org/licenses/by/4.0/).
